# Galactooligosaccharides and Resistant Starch Altered Microbiota and Short-Chain Fatty Acids in an *in vitro* Fermentation Study Using Gut Contents of Mud Crab (*Scylla paramamosain*)

**DOI:** 10.3389/fmicb.2020.01352

**Published:** 2020-06-30

**Authors:** Ngoc Tuan Tran, Yong Tang, Zhongzhen Li, Ming Zhang, Xiaobo Wen, Hongyu Ma, Shengkang Li

**Affiliations:** ^1^Guangdong Provincial Key Laboratory of Marine Biology, Shantou University, Shantou, China; ^2^Institute of Marine Sciences, Shantou University, Shantou, China; ^3^STU-UMT Joint Shellfish Research Laboratory, Shantou University, Shantou, China

**Keywords:** butyric acid, gut microbiota, prebiotic, *Scylla paramamosain*, short-chain fatty acids

## Abstract

Dietary carbohydrates are anaerobically fermented by gut microbiota to short-chain fatty acids (SCFAs), conferring gut health benefits. Of all tested prebiotics, galactooligosaccharides (GOS) and resistant starch (RS) stimulated the SCFA production in mud crab (*Scylla paramamosain*), a crustacean model, to a greater extent than the other carbohydrates tested. Using *in vitro* anaerobic fermentation cultures, this study further explored the prebiotic potential of GOS and RS in mud crab by assessing their impacts on gut microbiota changes and SCFA production. Both GOS and RS significantly promoted SCFA production. Bacterial diversity in the GOS group was lower than in the RS or control group. GOS promoted the growth of Bacteroidetes, while RS promoted Tenericutes. A strong positive correlation was found between SCFA production and bacterial abundance; most bacteria *per se* correlated with each other. The findings demonstrated the prebiotic potential of GOS and RS in mud crab.

## Introduction

The intestinal tract of aquatic animals is harbored by a large number of symbiotic microbes (Wang et al., 2018). Gut microbiota are important for host health and nutritional metabolism through multiple mechanisms (Li et al., 2018; Ringø et al., 2018). It has been found that, in aquatic animals, the composition of gut microbiota is influenced by several factors, including age, food type, feeding regimen of fish, as well as environmental and ecological factors (Ringø et al., 2006; Kashinskaya et al., 2014). Of these factors, the feeding regimen is one of the most important. In fact, consuming specific diets may affect the composition and activity of indigenous gut microbiota (Ringø et al., 2010). Prebiotics and non-digestible foods are some crucial components of diets that indirectly contribute to the health-promoting effects of the hosts (Gibson, 2004). In principle, prebiotics are known to promote the growth of beneficial bacteria, which stimulates metabolite production by gut microbes (Reichardt et al., 2018). In aquatic animals, short-chain fatty acids (SCFAs, including acetic, propionic, and butyric acids) produced by gut bacteria through anaerobic fermentation of dietary carbohydrates in the gut have been demonstrated to confer gut health benefits (Tran et al., 2020). The rate and amount of SCFA production depend on the species and amounts of gut microbiota, substrate sources, and gut transit time (Wong et al., 2006). Previously, some prebiotics such as α-starch, gelatinized starch, inulin, maize, barley, wheat, rye, lactosucrose, arabinoxylooligosaccharides, fructooligosaccharides, and pregelatinized tapioca starch were shown to affect the formation of SCFAs in the gut of aquatic animals *in vivo* (Tran et al., 2020).

Butyrate has been of great interest in human biology because of its roles in nourishing gut mucosa, preventing gut cancer by promoting cell differentiation, cell-cycle arrest, and apoptosis of transformed colonocytes, as well as inhibiting the enzyme histone deacetylase and decreasing the transformation of primary to secondary bile acids as a result of colonic acidification (Wong et al., 2006). Dietary supplementation of carbohydrates is a way to modulate microbial fermentation and promote butyrate production in the gut, which consequently improves health status (Sato et al., 2017). Recent studies in aquatic animals have demonstrated the roles of butyrate in improving growth performance, feed efficiency, immune responses, disease resistance, and survival rate, as well as in modulating gut microbiota (Gao et al., 2011; Robles et al., 2013; Liu B. et al., 2014; Estensoro et al., 2016; Rimoldi et al., 2016; Silva et al., 2016a, b; Terova et al., 2016; Ebrahimi et al., 2017; Piazzon et al., 2017; Ramírez et al., 2017; Tian et al., 2017; Ali et al., 2018; Rimoldi et al., 2018). For these reasons, the selection of a prebiotic capable of promoting the growth of butyrate-producing bacteria is necessary to enhance survival rate and improve aquaculture production. Supplementation with prebiotics might be a promising method that could confer positive health benefits in aquatic animals, including cultured crustaceans (Daniels et al., 2013; Chen et al., 2017; Duan et al., 2017; Elshopakey et al., 2018; Safari and Paolucci, 2018). *In vitro* anaerobic fermentation studies provide a primary approach for initial evaluation of the positive benefits of compounds through their effects on metabolic activities of gut microbiota (Pham et al., 2017). Many research efforts aimed at assessing the impacts of potential prebiotics on the dynamics of human gut microbiota have applied *in vitro* fermentation approaches (Scott et al., 2014; Pham et al., 2017; Sato et al., 2017; Baxter et al., 2019). These studies have demonstrated that prebiotics (i.e., L-sorbose, xylitol, and resistant starch) can selectively stimulate the growth of butyrate producers and increase the production of butyric acid in batch cultures. Accordingly, several substrates, such as arabinoxylan, whole wheat, soybean oligosaccharides, isomaltooligosaccharides, raffinose, gentiobiose, lactosucrose, arabinoxylanoligosaccharides, oligofructose, xylose, and fructose, have been shown to enhance the levels of SCFAs in batch cultures with gut microbes of common carp (*Cyprinus carpio*) (Kihara and Sakata, 2002), Nile tilapia (*Oreochromis niloticus*), and European seabass (*Dicentrarchus labrax*) (Leenhouwers et al., 2008), Siberian sturgeon (*Acipenser baerii*), and African catfish (*Clarias gariepinus*) (Geraylou et al., 2014). In this study, mud crab (*Scylla paramamosain*) was used as a model to investigate the effects of two kinds of carbohydrate (galactooligosaccharides [GOS] and resistant starch [RS]) on gut microbiota and SCFA production using *in vitro* anaerobic fermentation cultures. The results of this study provide an effective approach in the selection of prebiotic potential via stimulation of beneficial gut microbiota populations and the induction of metabolites.

## Materials and Methods

### Substrates and *in vitro* Fermentation

Two kinds of carbohydrates, including GOS and RS (manufactured by Yuanye Shengwu Co. Ltd., Shanghai, China) were investigated in this study. Both GOS and RS were demonstrated to stimulate the formation of much more butyric acid than the other kinds of carbohydrates tested (xylooligosaccharides, mannan-oligosaccharides, fructooligosaccharides, inulin, D-mannitol, D-sorbitol, L-sorbose, and xylitol) (Supplementary Text S1). The carbohydrate stock solutions were prepared in water, boiled for 1 min, and maintained anaerobically using O_2_-free N_2_. The saturated peptone-yeast extract (PY) (1 L) contained 5.0 g of peptone, 5.0 g of trypticase peptone, 10.0 g of yeast extract, 0.5 g of L-cysteine HCl⋅H2O, 4.0 g of Na_2_CO_3_, 10 mL of 0.05% hemin solution, 1.0 mL of 0.1% resazurin solution, 0.4 g of K_2_HPO_4_, 0.04 g of KH_2_PO_4_, 0.08 g of Na_2_HCO_3_, 0.04 g of NaCl, 8 mg of CaCl_2_, 1.9 mg of MgSO_4_⋅7H_2_O (pH 6.8), and 1 mg of vitamin K_1_ (Sato et al., 2017).

Gut contents were obtained from 18 healthy mud crabs (average weight 63.6 ± 8.8 g) without clinical disease purchased from a culture farm in Shantou (Guangdong, China). All mud crabs were randomly divided into three groups (of replications), chilled on ice, and then dissected. The intestinal tract was aseptically removed, and the contents from the posterior gut were gently squeezed out, placed into sterile Eppendorf tubes, and immediately transferred to an anaerobic (10% H_2_, 5% CO_2_, 85% N_2_) workstation (Whitley Workstation DG250, Don Whitley Scientific Ltd., Bingley, West Yorkshire, United Kingdom) upon delivery to carry out the experiment. Gut content samples were homogenized in a 10-fold dilution of anaerobic 0.1 M sodium phosphate buffer (pH 6.8) in a sterile Eppendorf tube. The mixtures (1 mL) were transferred to Hungate tubes sealed with butyl rubber stoppers and screw caps containing 4 mL of O_2_-free CO_2_-PY broth supplemented with 0.05 g of GOS or RS. Immediately after dilutions of the sample into tubes containing PY broth supplemented with carbohydrate, the mixture (2 mL) was collected from each test tube using a sterile syringe for time point 0 h. All steps for the cultures were conducted in an anaerobic chamber. Incubations for each carbohydrate and control (without carbohydrate supplementation) were performed in triplicate on a shaker (Shanghai Bluepard Instruments Co., Ltd.) at 140 × *g* for 24 h at 30°C. At 24 h after incubation, the samples being collected in the same manner. All samples collected were stored at −80°C until further analysis.

### Short-Chain Fatty Acid Analysis

Fermentation samples were thawed at room temperature and centrifuged at 10,000 × *g* for 10 min at 4°C. After centrifugation, the culture supernatants (1.0 mL) of the sample was removed and used for assessing SCFA production via gas chromatography after a 24-h growth as described previously (Li et al., 2019). The pH of the supernatant was adjusted to 2–3 by addition of H_2_SO_4_ and mixed thoroughly (2 min). The supernatant was collected after centrifugation at 13,000 × *g* for 10 min (at 4°C). The supernatant was collected and filtered (using a 0.2-μm nylon syringe filter). The filtrate was used for analyzing SCFA production using Agilent 6890N Network Gas Chromatograph (Agilent Technologies, Inc., Santa Clara, CA, United States) with a flame ionization detector equipped with a polar HP-INNOWAX capillary column (30 mm × 0.25 mm, 0.25 μm). A 1-μL injection volume of the sample was automatically injected into an inlet (at 230°C, 10:1 split ratio). Nitrogen was used as the carrier gas (flow rate: 1.0 mL/min). The detector temperature was 250°C, and the injector temperature was 230°C. The flow rates of nitrogen, hydrogen, and air as auxiliary gases were 25, 40, and 450 mL/min, respectively. The initial oven temperature was 50°C, which was maintained for 6 min and then raised to 230°C at 15°C/min and held at that temperature for 5 min.

Quantification of SCFAs in the fermentation products was performed using the calibration standard curves method. Three calibration standards were prepared at eight levels of concentration ranging from 0.699 to 69.9 mM for acetic acid and 0.218 to 21.8 mM for propionic and butyric acids. SCFA standards with three replicates were also treated as with the fermentation samples. From the chromatograms, the peak area of each calibration standard was plotted against the concentration of the SCFAs. Calibration curves were built using linear regression implemented in SPSS 16.0, after which the slope *a*, *y*-intercept *b*, and square correlation coefficients (*r*^2^) for the tested acids were calculated. Concentrations of acetic, propionic, and butyric acids were determined using the standard curve of each SCFA and expressed as mean mM.

### DNA Extraction, PCR Amplification, and Sequencing

Genomic DNA was extracted using the TIANamp Stool DNA Kit (Tiangen Biotech Co., Ltd., Beijing, China) according to the manufacturer’s instructions. The quality and quantity of extracted DNA were assessed using 2% agarose gel electrophoresis and a NanoDrop 2000 spectrophotometer. The DNAs were stored at −20°C until use. The V4 region of the 16S rRNA gene was amplified using barcoded universal primers: 515F (5′-GTGCCAGCMGCCGCGGTAA-3′) and 806R (5′-GGACTACHVGGGTWTCTAAT-3′) (Caporaso et al., 2011) with the barcode. Polymerase chain reaction (PCR) reactions were conducted as described previously (Li et al., 2019). Briefly, each PCR reaction was carried out in a 30-μL volume containing 10 ng of DNA, 15 μL of Phusion^®^ High-Fidelity PCR Master Mix with GC buffer (New England Biolabs, Ipswich, MA, United States), 0.2 μM forward and reverse primers, and 2 μL of distilled water. The PCR conditions were as follows: 1 min of initial denaturation at 98°C; 30 cycles of 98°C for 10 s, 50°C for 30 s, and 72°C for 30 s; and a final elongation step at 72°C for 5 min. The PCR products were separated using agarose gel electrophoresis and purified by application of a DNA Gel Extraction Kit (Axygen, Hangzhou, China). The concentration of the purified DNA was determined on a NanoDrop 2000 spectrophotometer. Sequencing libraries were synthesized using Ion Plus Fragment Library Kit 48 rxns (Thermo Fisher Scientific, Waltham, MA, United States) and assessed on a Qubit^®^ 2.0 Fluorometer (Thermo Fisher Scientific). The libraries were sequenced on an IonS5^TM^XL platform (Thermo Fisher Scientific) and 400-bp single-end reads were generated.

### Bioinformatics and Data Analysis

All sequence reads were sorted based on their unique barcodes. The sequence quality was filtered using Cutadapt 1.9.1 (Martin, 2011). Chimera sequences were removed using the UCHIME algorithm (Edgar et al., 2011). Sequences were clustered into operational taxonomic units (OTUs) using 97% identity as the cut-off value by Uparse software (Version 7.0.1001) (Edgar, 2013). The representative sequences from each OTU were taxonomically classified using the SILVA reference database with a confidence threshold of 80%. The alpha diversity indices, richness estimators (Chao1 and Abundance-based Coverage Estimator [ACE]), and diversity index (Simpson, Shannon index) were all calculated using QIIME 1.7.0 (Caporaso et al., 2010). For the beta diversity metrics, principal coordinates analysis (PCoA) was applied using the UniFrac web tool (Lozupone et al., 2006). Bacterial community profiling data were analyzed statistically with one-way analysis of variance (ANOVA) implemented in SPSS 16.0. Non-parametric permutational multivariate analysis of variance (PERMANOVA) was performed via PAST 2.16 (Hammer et al., 2001) to evaluate the significance of differences in bacterial community structure among groups. The association between the SCFA production and microbiota community was analyzed using SPSS 16.0. Differences obtained by these tests were considered statistically significant at *P* < 0.05. Heat maps showing the correlation between the relative abundance of microbial taxa and SCFA production were generated via Heml 1.0^1^. Linear discriminant analysis effect size (LEfSe) analysis to determine microbial genera with significantly different abundances between groups was performed using Galaxy/Hutlab^2^.

## Results

### Potential Prebiotics and SCFA Analysis

**FIGURE 1:**
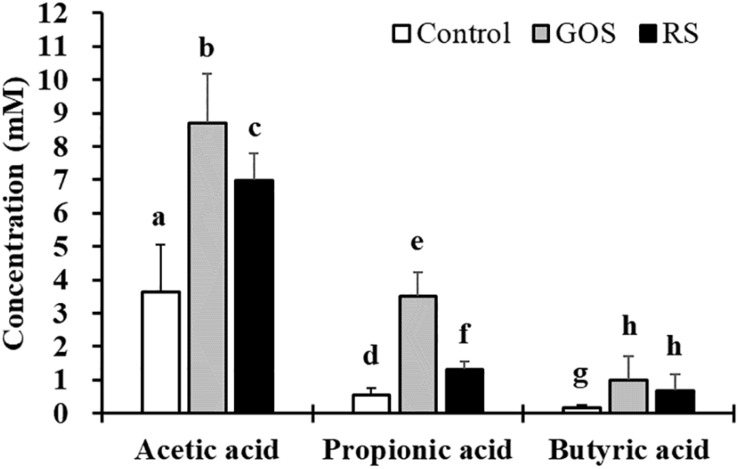
Production of short-chain fatty acids (SCFAs) in the *in vitro* fermentation of galactooligosaccharides (GOS) and resistant starch (RS) with gut contents of mud cab. Different letters indicate significant differences between groups within each kind of SCFAs (*P* < 0.05).

To investigate the effects of GOS and RS on the metabolic activities of gut microbiota in mud crab, an *in vitro* anaerobic fermentation study was applied. The concentrations of SCFAs in cultures supplemented with either GOS or RS estimated at 0 and 24 h are shown in Figure 1. The results revealed that, compared with control, both GOS and RS significantly increased the concentrations of acetic acid (2.4- and 1.9-fold, respectively), propionic acid (6.3- and 2.3-fold, respectively), and butyric acid (6.4- and 4.3-fold, respectively) (*P* < 0.05). In addition, the concentrations of both acetic acid and propionic acid were significantly higher in the GOS compared with that in RS (*P* < 0.05), while that of butyric acid was not significantly different between the two groups (*P* = 0.29).

### Microbial Sequencing and Community Structure

**FIGURE 2:**
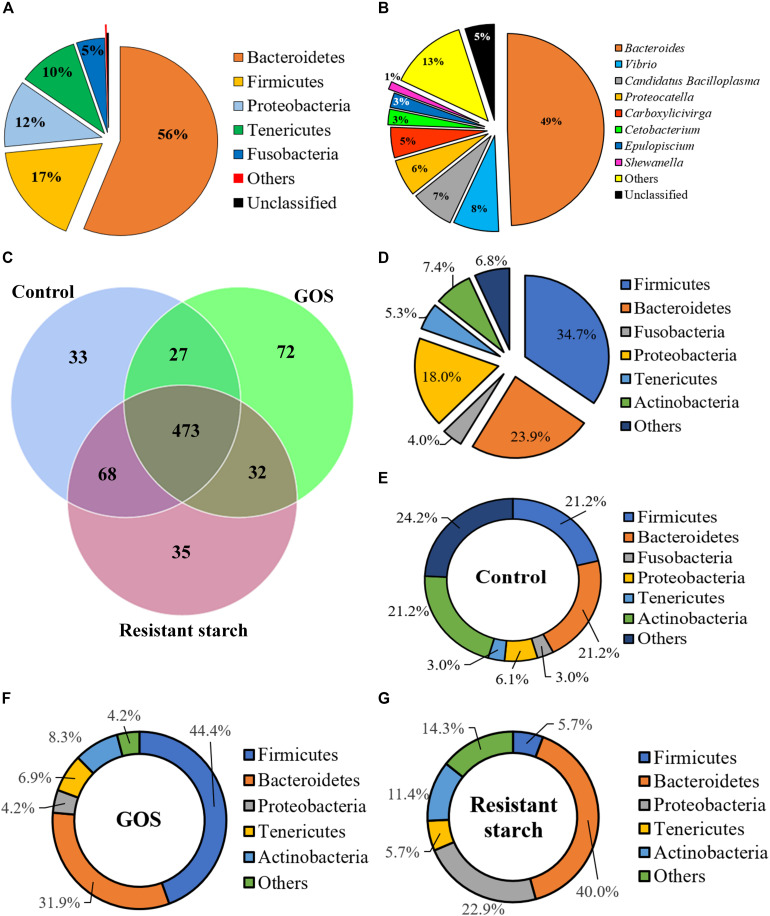
The composition of gut microbiota of galactooligosaccharides and resistant starch (using the combined data set) and the unique and shared OTUs in the cultures after a 24-h *in vitro* fermentation. **(A)** The five high-abundance phyla combined accounted for 99.53% of all OTUs, whereas low-abundance phyla accounted merely for 0.19% (marked as “others”) and 0.28% (“unclassified”). **(B)** The five genera with abundance ≥ 1% accounted for 81.92% of the total microbiota; the sequences that could not be classified into any known genus were marked as “unclassified” (accounting for 5.0%). **(C)** Venn diagram shows the number of shared and unique OTUs among the samples in different groups (the phylum level). **(D–G)** Pie charts show the characteristics of shared OTUs **(D)** and unique OTUs in the samples in controls **(E)**, GOS **(F)**, and resistant starch **(G)** groups (at the phylum level).

To investigate the influence of GOS or RS on gut microbial community structure, high-throughput sequence analysis of bacterial hypervariable V4 region of the 16S rRNA gene was performed. Raw sequencing reads have been deposited in GenBank under BioProject code PRJNA565751, BioSample numbers SAMN12767047, SAMN12767049, and SAMN12767050. After quality filtering and chimera removal, the 27 samples (9 for each group) yielded a total of 2,027,455 high-quality reads, with an average number of 75,090.93 reads per sample. The rarefaction curves reached the saturation plateau (Supplementary Figure S1A), indicating that saturation in sequencing was obtained. The rank abundance curves revealed a similarity in diversity in all samples (Supplementary Figure S1B). The Good’s coverage for each sample ranged from 99.9 to 100%, indicating that most of the bacteria present in the samples were identified (Supplementary Figure S1C). The reads were assigned to 741 OTUs at a 97% sequence identity threshold. Each sample contained 277–436 OTUs. Across all the samples, 97.98 and 63.83% of the total sequences were assigned into 18 phyla (control: 16; GOS: 15; RS: 16) (Supplementary Table S1) and 144 genera (control: 101; GOS: 90; RS: 108), respectively (Supplementary Table S2). At the phylum level, the samples predominantly consisted of five phyla: Bacteroidetes, Firmicutes, Proteobacteria, Tenericutes, and Fusobacteria, which together accounted for 99.53% of the OTUs (Figure 2A). Specifically, the genera *Bacteroides*, *Vibrio*, *Candidatus Bacilloplasma*, *Proteocatella*, *Carboxylicivirga*, *Cetobacterium*, *Epulopiscium*, and *Shewanella* were the most abundant gut microbiota (Figure 2B). All these genera together accounted for 81.92% of the total microbiota.

A Venn diagram revealed the numbers of unique or common OTUs (Figures 2C–G). Figure 2C showed that the numbers of unique OTUs in the control, GOS, and RS groups were 33, 72, and 35, respectively, and the numbers of shared OTUs among groups were 473 OTUs. The GOS and control samples shared 27 OTUs, while the RS and controls shared 68 OTUs and the GOS and RS groups shared 32 OTUs. At the phylum level, the most abundant unique and shared OTUs were determined as Firmicutes, Bacteroidetes, Proteobacteria, Tenericutes, Actinobacteria, and Fusobacteria (Figures 2D–G).

### Microbial Diversity

**FIGURE 3:**
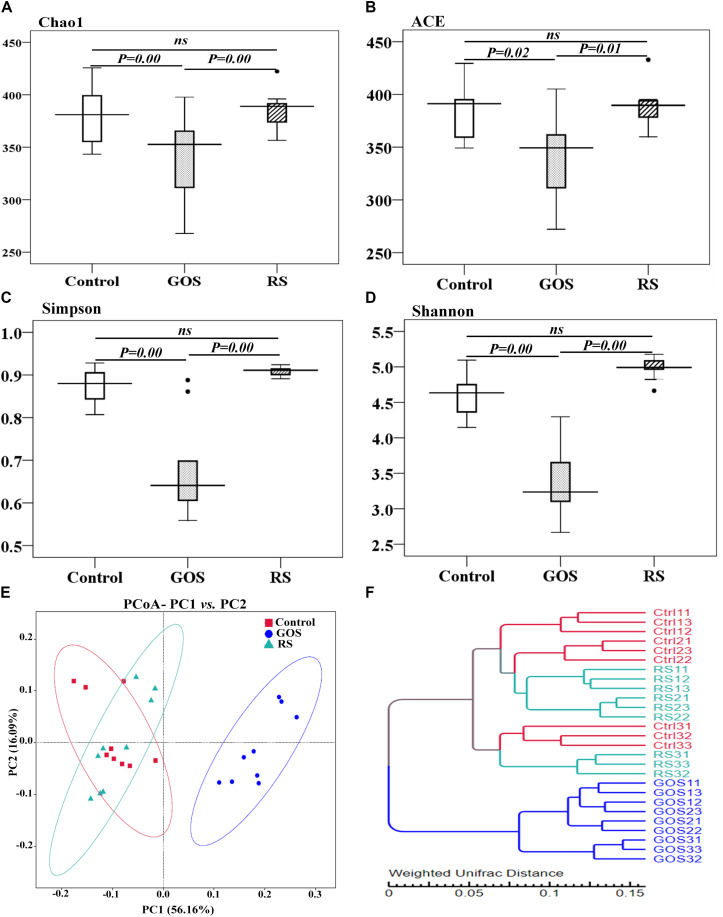
Alpha diversity estimators and bacterial community composition of gut microbiota populating the cultures after a 24-h *in vitro* fermentation of GOS or RS. **(A–D)** Alpha diversity estimators: Chao1 **(A)**, ACE **(B)**, Simpson **(C)**, and Shannon **(D)**. Differences are determined by ANOVA Turkey test (with *P* < 0.05). A non-significant difference is indicated with “ns.” **(E)** Principal coordinate analysis (PCoA) (PC1: 56.16% and PC2: 16.09% of the explained variance). **(F)** Cluster analysis using a weighted pair-group method with arithmetic mean. Each dot shows a single sample (Ctrl, GOS, and RS indicate the samples from the control, galactooligosaccharide, and resistant starch groups, respectively).

The microbial complexity in the three groups was estimated using alpha diversity indices, including richness estimators (Chao1 and ACE) and diversity indexes (Simpson and Shannon index) (Figures 3A–D). Statistical testing revealed that the alpha diversity indices were significantly different between the GOS and controls or RS (*P* < 0.05), but not between RS and controls (*P* > 0.05). The beta diversity analysis, as estimated by Turkey and Wilcoxon tests, was significantly different between control and GOS (*P* = 0.00 and 0.00, respectively) or GOS and RS (*P* = 0.00 and 0.00, respectively), but not between control and RS (*P* > 0.05). The PERMANOVA analysis revealed a significant difference in overall bacterial community structure using the weighted UniFrac distance matrix between GOS and control (*F* = 53.25, *P* = 0.0001), GOS and RS (*F* = 55.7, *P* = 0.0002), and RS and control (*F* = 1.128, *P* = 0.331). A principal coordinate analysis (PCoA) (based on weighted UniFrac distance matrixes) was used to estimate the similarity in the microbial community composition of the samples (Figure 3E). A scatterplot based on PCoA scores revealed a difference in the bacterial community composition in the GOS in comparison to the RS and control and more similarity in that composition between the RS and control groups. This observation was also found in the weighted pair-group method with arithmetic means (WPGMA) clustering analysis (Figure 3F). The samples from the GOS group tend to cluster together, whereas the RS and control seemed to be more variable and grouped together.

### Microbial Composition

**FIGURE 4:**
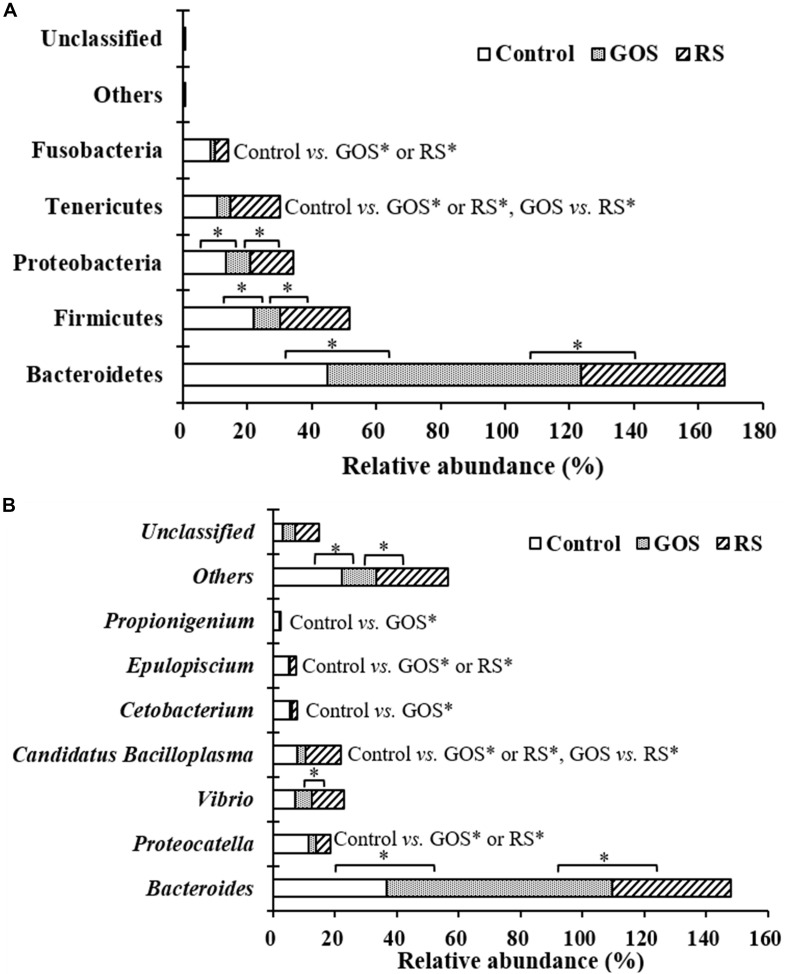
Composition of gut microbiota at the phylum **(A)** and genus **(B)** levels in the cultures after a 24-h *in vitro* fermentation of galactooligosaccharides (GOS) and resistant starch (RS), as well as in the controls. Differences determined by ANOVA Turkey test (with *P* < 0.05) are indicated with an asterisk.

Differences in the taxonomical profiles [(Supplementary Table S1) and genus (Supplementary Table S2) levels] were analyzed. At the phylum level, Bacteroidetes, Firmicutes, Proteobacteria, Tenericutes, and Fusobacteria were predominant (together accounting for 99.4, 99.8, and 99.4% of the microbiota in the control, GOS, and RS, respectively). After 24 h of incubation with GOS or RS, the relative abundance of the dominant phyla changed (Supplementary Table S1 and Figure 4A). Compared to the control, GOS significantly increased the levels of Bacteroidetes and significantly decreased those of Firmicutes, Proteobacteria, Tenericutes, and Fusobacteria (*P* < 0.05). Also, the relative abundance of Bacteroidetes was significantly increased in the GOS compared to the RS, while that of Firmicutes, Proteobacteria, and Tenericutes was significantly decreased (*P* < 0.05). In the RS group, compared with control, the most notable changes were a significant increase in the levels of Tenericutes and a significant decrease in that of Fusobacteria (*P* < 0.05). Nevertheless, there was no significant difference in the remaining phyla (*P* > 0.05).

At the genus level, the most abundant genera were classified as *Bacteroides*, *Carboxylicivirga*, *Proteocatella*, *Vibrio*, *Shewanella*, *Candidatus Bacilloplasma*, *Cetobacterium*, *Epulopiscium*, and *Propionigenium* (together accounting for 84.40, 89.32, and 74.60% of the microbiota in the control, GOS, and RS groups, respectively) (Figure 4B). After 24 h of incubation, the relative abundance of 25 of 144 classified genera changed across samples among the three groups (*P* < 0.05) (Supplementary Table S2). GOS dramatically increased the levels of *Bacteroides* and significantly decreased those of *Proteocatella*, *Candidatus Bacilloplasma*, *Cetobacterium*, *Epulopiscium*, and *Propionigenium* compared with the controls (Figure 4B). *Bacteroides* became more abundant, while *Vibrio* and *Candidatus Bacilloplasma* became less abundant in the GOS group compared to the RS (Figure 4B). In the RS group, compared to the controls, a significant increase in *Candidatus Bacilloplasma* abundance and a significant decrease in *Proteocatella* and *Epulopiscium* abundance were observed (Figure 4B).

**FIGURE 5:**
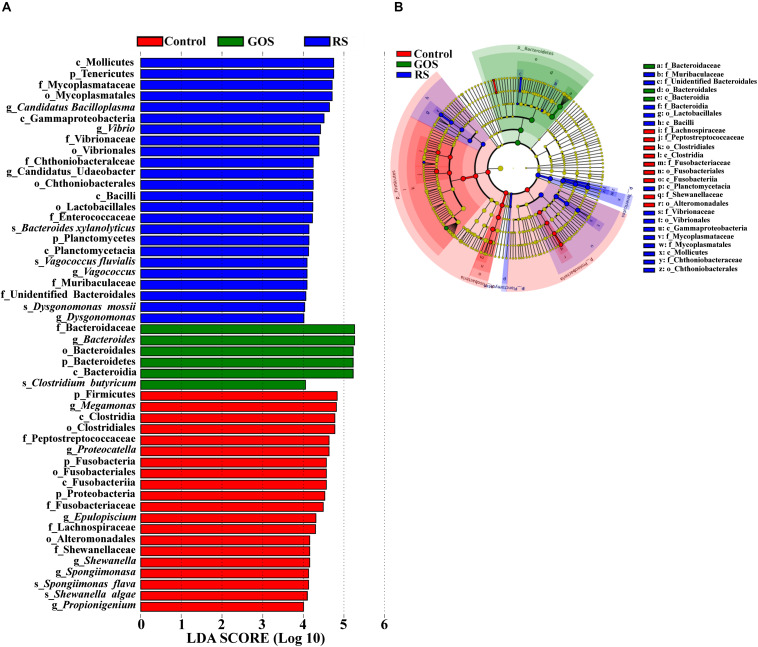
Characterization of gut microbiota in the cultures after a 24-h *in vitro* fermentation of GOS and RS, as well as in the controls by LDA and LEfSe analysis. **(A)** Histogram of the LDA scores (log10) calculated for features differentially abundant in control, GOS, and RS samples (with LDA scores > 4.0). **(B)** Bacterial taxa differentially represented among groups identified by LEfSe.

Linear discriminant analysis (LDA) effect size (LEfSe) analysis (with LDA scores > 4.0) was performed to determine the specific taxa responsible for the differences among the three populations (Figure 5). A total of 50 bacterial taxa (24 in control; 6 in GOS; 20 in RS) showed differences among three populations (Figure 5A). Firmicutes and Fusobacteria were abundant in the control, while Bacteroidetes was abundant in the GOS, and Tenericutes and Proteobacteria in the RS group. Specifically, *Bacteroides* and *Clostridium butyricum* were abundant in the GOS, whereas *Candidatus Bacilloplasma*, *Vibrio*, *Vagococcus*, *Dysgonomonas*, *Bacteroides xylanolyticus*, *Vagococcus fluvialis*, and *Dysgonomonas mossii* were abundant in the RS. The cladogram analysis also revealed differences in bacterial taxa among the experimental groups (Figure 5B).

### Associations Between Gut Microbiota and SCFAs

**FIGURE 6:**
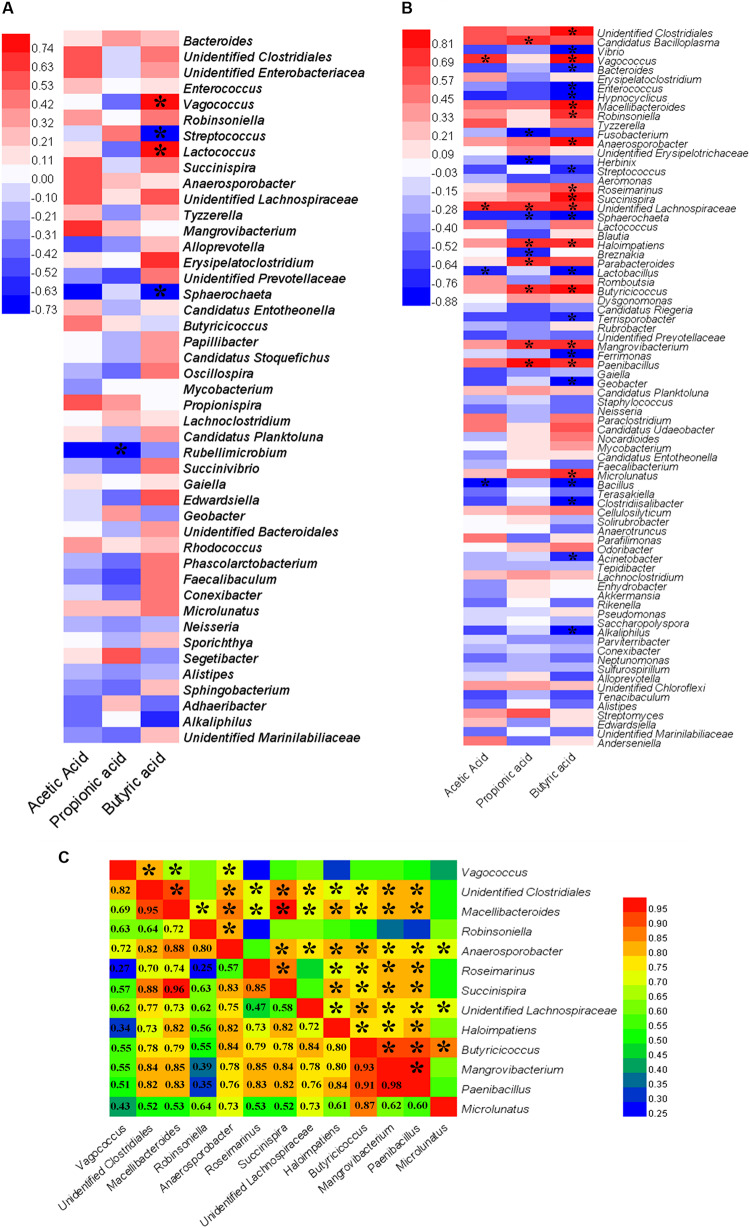
A heat map of Spearman’s correlation coefficients. **(A,B)** Correlation between the abundance of key microbial taxa (at the genus levels) and the production of short-chain fatty acids (SCFAs, including acetic, propionic, and butyric acids) in the cultures after a 24-h *in vitro* fermentation of galactooligosaccharides **(A)** and resistant starch **(B)**. Red and blue colors indicate positive and negative correlation coefficients, respectively. Significant correlations (*P* < 0.05) are indicated with an asterisk. **(C)** Correlation among the bacterial genera that had a significantly positive correlation with the production of butyric acid in the cultures after a 24-h *in vitro* fermentation of resistant starch. Numbers indicate Spearman’s correlation coefficients. Significant correlations (*P* < 0.05) are indicated with an asterisk.

Spearman’s correlation analysis was used to determine the relationships between differentially abundant taxa (at the genus levels) and SCFA production (Figures 6A,B). In the GOS group, butyric acid was positively correlated with the genera *Vagococcus* (Spearman’s ρ [rs] = 0.79, *P* = 0.01) and *Lactococcus* (rs = 0.84, *P* = 0.00) (Figure 6A). In the RS group (Figure 6B), acetic acid had a significant positive correlation with *Vagococcus* (rs = 0.70, *P* = 0.04) and unidentified Lachnospiraceae (rs = 0.72, *P* = 0.03). Propionic acid was found to be strongly positively correlated with *Candidatus Bacilloplasma* (rs = 0.75, *P* = 0.02) and *Parabacteroides* (rs = 0.71, *P* = 0.03). Furthermore, a strong positive correlation was observed between butyric acid and *Vagococcus* (rs = 0.83, *P* = 0.01), *Macellibacteroide* (rs = 0.88, *P* = 0.00), *Robinsoniella* (rs = 0.74, *P* = 0.02), *Anaerosporobacter* (rs = 0.88, *P* = 0.00), *Roseimarinus* (rs = 0.68, *P* = 0.05), *Succinispira* (rs = 0.85, *P* = 0.00), *Haloimpatiens* (rs = 0.71, *P* = 0.03), *Butyricicoccus* (rs = 0.84, *P* = 0.00), *Mangrovibacterium* (rs = 0.78, *P* = 0.01), *Paenibacillus* (rs = 0.76, *P* = 0.02), *Microlunatus* (rs = 0.73, *P* = 0.03), unidentified Clostridiales (rs = 0.93, *P* = 0.00), and unidentified Lachnospiraceae (rs = 0.73, *P* = 0.03).

### Correlations Among Bacteria Related to Butyric Acid Production

The correlations among bacterial taxa that had a significant positive correlation with the production of butyric acid were estimated. The results revealed that *Vagococcus* had a significant positive correlation with *Lactococcus* (rs = 0.93, *P* = 0.00) in the GOS. In the RS group, most genera were significantly positively correlated with others (*P* < 0.05) (Figure 6C). For example, *Butyricicoccus* were positively correlated with other taxa (rs > 0.55, *P* < 0.05), except *Vagococcus* (rs = 0.55, *P* = 0.12). The current findings indicated that the supplemented carbohydrates were fermented not only by a bacterium (or bacterial genus), but also by a population of bacteria to produce butyric acid.

## Discussion

Dietary carbohydrate intake could influence the composition of gut microbiota and its metabolic products in the host intestinal tract (Scott et al., 2014). In aquatic animals, it has been shown that dietary carbohydrates can selectively promote the growth of beneficial microbes (including SCFA producers) inducing gut fermentation and SCFA formation (Tran et al., 2020). In this study, mud crab was used as a model organism to explore the effects of potential prebiotics (GOS and RS) selected from 10 kinds of carbohydrates on microbial composition and production of SCFAs under anaerobic fermentation with gut content samples in batch cultures. The production of SCFAs was measured using gas chromatography analysis while the composition of microbiota was determined using 16S rRNA sequencing.

SCFAs are the predominant end-products of bacterial fermentation of indigestible dietary carbohydrates, which confer beneficial health outcomes to the host (Piazzon et al., 2017; Tran et al., 2020). SCFAs are readily and quickly absorbed by host gut colonocytes, limiting the use of animal models; hence, *in vitro* fermentation cultures are useful for studying SCFA formation during fermentation (Inness et al., 2011). In this study, the concentrations of acetic, propionic, and butyric acids were significantly increased in batch cultures supplemented with either GOS or RS, indicating that both substrates could be fermented by microbes, as evidenced by SCFA production. This is similar to the results of a previous study in which cereal grains were used to stimulate the production of volatile fatty acids in Nile tilapia (*O. niloticus*) and European seabass (*D. labrax*) (Leenhouwers et al., 2008). Also, the supplementation of oligosaccharides increased the levels of total SCFA (sum of acetic, propionic, and *n*-butyric acids) in batch cultures with the gut contents of common carp (*C. carpio*) (Kihara and Sakata, 2002). Butyric acid shows protective effects, such as improving epithelial barrier function, gut permeability, and gut immune functions, on animals (Liu W. et al., 2014; Rimoldi et al., 2016; Safari et al., 2016; Piazzon et al., 2017; Tian et al., 2017). The term “prebiotics” has been defined as “ingredients of the non-digestible diet that is beneficial to the host for stimulating selectively the growth and/or the activity of one or more intestinal bacteria” (Gibson, 2004). Thus, a substrate that selectively stimulates the growth of butyrate-producing bacteria and is fermented to butyric acid meets the prebiotic definition criterion. It was observed that no significant difference in the concentration of butyric acid was found between the GOS and RS groups (*P* > 0.05), but a significant increase was observed between either GOS or RS and control (*P* < 0.05). The findings here indicate the prebiotic potential of GOS and RS in mud crab. Consistent with this view, it has been previously suggested that GOS and starch are potential prebiotics that affect growth performance, gut microbiota, and immune response in fish species, *in vivo* (Kihara and Sakata, 1997; Amirkolaie et al., 2006; Hoseinifar et al., 2013, 2017; Miandare et al., 2016; Romano et al., 2018). The effects of prebiotic-driven bacteria present in inocula on the relative proportions and rates of SCFAs produced in batch cultures have previously been reported (Sato et al., 2017; Reichardt et al., 2018; Baxter et al., 2019). Thus, this evidence supports the prebiotic potential of GOS and RS in modulating gut microbiota, which may indirectly improve the immune responses in mud crab. However, this needs to be confirmed in further studies.

Apart from the production of SCFAs from fermentation, prebiotics are expected to have the capacity to selectively promote the growth and/or activity of gut bacteria involved in health and well-being (Pham et al., 2017). Changes in the community composition due to the ingestion of dietary fibers occur as a result of differences in their physicochemical and metabolic properties (Umu et al., 2015). As shown in the Venn diagram, all samples in the control, GOS, and RS groups shared a large core of microbiota. This is consistent with previous findings in which gut microbiota of fish species on different trophic levels shared a large core microbiome (Liu et al., 2016), which might be important in the stability of the gut microbiota. In spite of this, each group still had a unique microbiome, suggesting that the supplemented carbohydrates mainly contributed to the growth of gut microbiota. Furthermore, the results of this study clearly revealed that GOS and RS induced shifts in the composition of gut microbiota, which correlated with the production of butyric acid in the batch cultures. These results are in agreement with previous studies, in which anaerobic fermentation with human fecal samples supplied with prebiotics (i.e., L-sorbose, xylitol, type 2 RS) was carried out *in vitro* (Scott et al., 2014; Pham et al., 2017; Sato et al., 2017; Reichardt et al., 2018; Baxter et al., 2019). Our data revealed that alpha diversity was lower in the GOS group than that in RS and the controls, which may be due to the selection of particular microbial communities within the prebiotic-supplemented samples. Consistent with this view, studies in young healthy humans treated with GOS for 14 days revealed worsened alpha diversity compared with those on day 0 (Liu et al., 2017). This supports the view that a more diverse community is coupled with a healthier and more stable ecosystem (Xiao et al., 2016). The decreased bacterial diversity, as shown in this study, may be explained by the carbohydrate-driven lowering of pH, which affects the survival capacity of acid-sensitive species and stimulates the overgrowth of acid-tolerant species, thereby suppressing bacterial diversity in batch cultures (Gross et al., 2010). Low pH (5.5) increased the growth of butyrate-producing bacteria, resulting in the elevation of butyric acid production (Reichardt et al., 2018). The results further support the selective stimulation of GOS on the growth of SCFA-producing bacteria. Notably, supplementation with RS led to an unchanged bacterial diversity in the batch cultures compared to the controls, indicating that the microbes were relatively constant, resulting in the stability of the living ecosystem on RS supplementation (Xiao et al., 2016). In contrast to our findings, alpha diversity within the microbiome was lower in pigs fed an RS diet compared to controls (Umu et al., 2015). The differences in results might be due to differences in experimental designs (*in vitro* vs. *in vivo*), study subjects, and microbial composition communities. Furthermore, the analyses of PERMANOVA, PCoA, and WPGMA clustering showed significant differences in beta diversity between the GOS group and RS or control (*P* < 0.05), suggesting that the diversity of gut microbiota in the batch cultures was affected by the supplemented carbohydrates.

Although the presence of bacterial communities (at the phylum level, with a dominance of Bacteroidetes, Firmicutes, Proteobacteria, Tenericutes, and Fusobacteria) was almost similar among the groups, the abundance of some phyla in the GOS and RS groups differed significantly from the controls. The composition of predominant bacterial phyla in this study was similar to those previously described for mud crab (*S. paramamosain*) (Li et al., 2012). This indicates that these bacterial members may be a core microbiome that harbors the gut of mud crab, conserving the stability of gut microbiota even when stimulated with supplemented carbohydrates under *in vitro* fermentation cultures. The increased level of the genera *Bacteroides* (GOS group) and *Candidatus Bacilloplasma* (RS group) suggests that the members of these genera may be important in degrading GOS and RS, respectively, in the batch cultures.

The benefits of butyric acid in the gut health of aquatic animals have been discussed previously (Tran et al., 2020). Remarkably, the relative proportions and rates of SCFAs produced are influenced by the bacteria present in the batch cultures (Inness et al., 2011). Herein, we tested whether the relative abundance of gut microbiota was related to the production of SCFAs through fermentation of the supplemented carbohydrates. In the GOS group, no bacterial genus was found to be associated with the production of acetic and propionic acids, suggesting that GOS herein is not an ideal substrate fermented by acetate- and propionate-producing bacteria. However, RS could be fermented by members of *Vagococcus* and unidentified Lachnospiraceae to acetic acid, as well as *Candidatus Bacilloplasma* and *Parabacteroides* to propionic acid. Interestingly, more bacterial genera were associated with the production of butyric acid in either the GOS or RS group. This finding supports the foregoing conclusions that GOS and RS are able to selectively promote the growth of butyrate producers, resulting in an increase of butyric acid production. Generally, the increased relative abundance of a bacterial taxon is inadequate to explain the association with butyrate production within samples (Baxter et al., 2019). Thus, the correlations among the bacterial genera that positively associated with the increased level of butyric acid within each group (GOS or RS) were investigated. Our results showed a significant positive correlation among the bacterial genera. This is similar to the results in a previous study that the correlation between *Ruminococcus bromii* and *Eubacterium rectale* was coupled with higher butyrate concentration in humans consuming unmodified starches (Baxter et al., 2019). The findings herein suggest that the carbohydrate-driven bacteria can interact syntrophically with others and change the metabolic pathways, which stimulated the production of butyric acid.

## Conclusion

**FIGURE 7:**
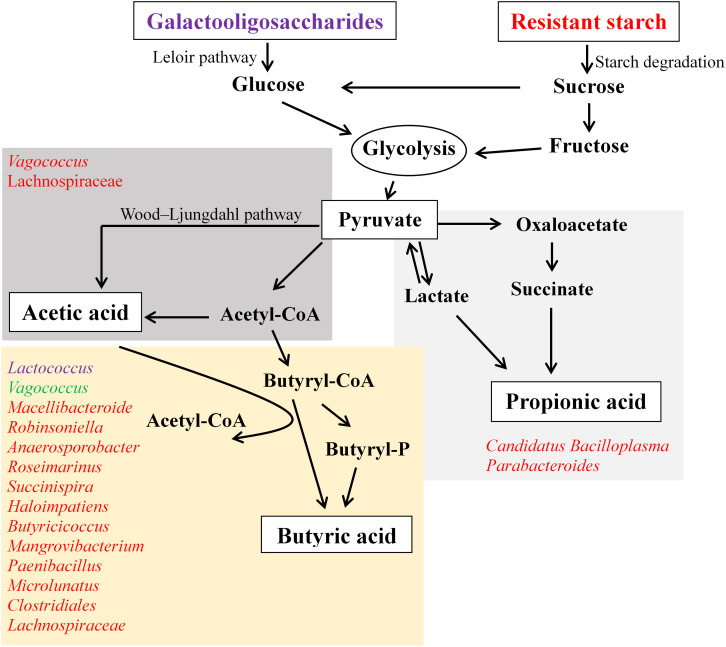
An overview of the production of acetic, propionic, and butyric acids in the *in vitro* fermentation of galactooligosaccharides (GOS) and resistant starch (RS) with gut contents of mud cab. Bacterial genera involved in the production of SCFAs were determined based on the Spearman’s correlation coefficients between the abundance of microbial taxa and the production of each kind of SCFA. Bacteria illustrated in purple indicate the correlations found in the GOS group, red indicates the correlations in the RS group, and green indicates the correlations in both GOS and RS groups.

This *in vitro* fermentation study provides insight into the supplementation of GOS and RS, which is capable of altering gut microbiota and the production of SCFAs. Generally, GOS induced a decrease in the diversity of gut microbial communities compared with RS and control, while RS did not change the diversity of these compared with controls. An overview of the microbiota related to the pathways for SCFA production in response to the supplementation of GOS or RS is shown in Figure 7. The production of butyric acid was found to be related to the increased relative abundance of many bacterial genera in both GOS and RS groups. Most of these genera were positively correlated with others, suggesting that the carbohydrate-driven bacteria could interact syntrophically with others and change the metabolic pathways to produce butyric acid. As the current study was conducted using only an *in vitro* fermentation approach, the results need to be confirmed by an *in vivo* mud crab fermentation study.

## Data Availability Statement

The datasets presented in this study can be found in online repositories. The names of the repository/repositories and accession number(s) can be found in the article/Supplementary Material.

## Ethics Statement

The animal study was reviewed and approved by all animal handling procedures in this study were reviewed and approved by the ethics committee of “The Regulations for The Administration of Affairs Concerning Experimental Animals.”

## Author Contributions

NT and SL designed the study, analyzed the data, and wrote the manuscript. YT, ZL, and MZ attended the sample processing, DNA isolation, and sequencing of samples. XW and HM reviewed the manuscript and provided the guidance. All the authors read and approved the final manuscript.

## Conflict of Interest

The authors declare that the research was conducted in the absence of any commercial or financial relationships that could be construed as a potential conflict of interest.

## Footnotes

1 http://hemi.biocuckoo.org/down.php

2 https://huttenhower.sph.harvard.edu/galaxy/

## Supplementary Material

FIGURE S1 Alpha diversity in rarefaction curves plot (A) and in rank abundance curves.

TABLE S1 Bacterial composition of gut microbiota in the batch cultures after 24 h of fermentation of galactooligosaccharides (GOS) and resistant starch (RS) (at the phylum level).

TABLE S2 Bacterial composition of gut microbiota in the batch cultures after 24 h of fermentation of galactooligosaccharides (GOS) and resistant starch (RS) (at the genus level).

TEXT S1 Prescreening of potential prebiotics and SCFA analysis.
