# Supplementary material for: Galactooligosaccharides and Resistant Starch Altered Microbiota and Short-Chain Fatty Acids in an in vitro Fermentation Study Using Gut Contents of Mud Crab (Scylla paramamosain)
Source: Front Microbiol. 2020 Jun 30;11:1352. doi: 10.3389/fmicb.2020.01352 (PMC7338486; doi:10.3389/fmicb.2020.01352)
Supplement: FIGURE S1 — Alpha diversity in rarefaction curves plot (A) and in rank abundance curves. [file Data_Sheet_2.docx]

**Figures S**

**FIGURE S1** Alpha diversity in rarefaction curves plot (A) and in rank abundance curves plot (B) and (C) the Good’s coverage for each sample.
