# Supplementary material for: Galactooligosaccharides and Resistant Starch Altered Microbiota and Short-Chain Fatty Acids in an in vitro Fermentation Study Using Gut Contents of Mud Crab (Scylla paramamosain)
Source: Front Microbiol. 2020 Jun 30;11:1352. doi: 10.3389/fmicb.2020.01352 (PMC7338486; doi:10.3389/fmicb.2020.01352)
Supplement: TEXT S1 — Prescreening of potential prebiotics and SCFA analysis. [file Data_Sheet_1.docx]

**TEXT S1** Prescreening of potential prebiotics and SCFA analysis

**Substrates, Gut Content Samples, and Cultures**

Ten kinds of carbohydrates, including galactooligosaccharides (GOS), xylooligosaccharides (XOS), mannan-oligosaccharides (MOS), fructooligosaccharides (FOS), inulin, D-mannitol, D-sorbitol, L-sorbose, xylitol, and resistant starch (RS), used in this study were purchased from the Yuanye Shengwu company (Shanghai, China). The carbohydrate stock solutions were prepared in water and boiled for 1 min and maintained anaerobically using O_2_-free N_2_. The saturated peptone-yeast extract (PY) (1 L) contained 5.0 g peptone, 5.0 g trypticase peptone, 10.0 g yeast extract, 0.5 g L-cysteine HCl.H2O, 4.0 g Na_2_CO_3_, 10 mL 0.05% hemin solution, 1.0 mL 0.1% resazurin solution, 0.4 g K_2_HPO_4_, 0.04 g KH_2_PO_4_, 0.08 g Na_2_HCO_3_, 0.04 g NaCl, 8 mg CaCl_2_, 1.9mg MgSO_4_.7H_2_O (pH 6.8) and 1 mg vitamin K1) ([Sato et al., 2017](#_ENREF_2)).

Gut contents obtained from healthy mud crabs (Culture I: 14 individuals with an average weight of 94.8±12.5 g; Culture II: 23 individuals with an average weight of 89.2±25.4 g), which were purchased from a culture farm in Shantou (Guangdong, China). All mud crabs (of each culture time) were divided into three groups (of replications), chilled on ice, and then dissected. Intestinal tract was aseptically removed, and the gut contents were gently squeezed out, placed into sterile Eppendorf tubes, and immediately transferred to an anaerobic (10% H_2_, 5% CO_2_, 85% N_2_) workstation (Whitley Workstation DG250, Don Whitley Scientific Ltd., UK) upon delivery to carry out the experiment. Gut content samples homogenized in 10-fold dilution of anaerobic 0.1 M sodium phosphate buffer (pH 6.8) in a sterile Eppendorf tube. The mixtures (Culture I: 100 μL; Culture II: 700 μL) were transferred into Hungate tubes sealed with butyl rubber stoppers and screw caps containing 5 mL of O_2_-free CO_2_-PY broth supplemented with 0.05 g of different LDCs. All steps for the cultures were conducted in an anaerobic chamber. Incubations for each NDC and control (without NDC supplementation) were performed in triplicate on a shaker (Shanghai Bluepard Instruments Co., Ltd.) at 140 rpm for 24 h at 30°C. At 0 h and 24 h, a volume of 2 mL was taken out from each test tube using a sterile syringe. All samples collected were stored at -80^o^C until further analysis. The butyric acid production after a 24-h growth was estimated using gas chromatography as described previously ([Li et al., 2019](#_ENREF_1)).

**The Formation of Butyric Acid in Gut Content Cultures**

In the pre-screening, the amounts of butyric acid produced from 10 kinds of carbohydrates were firstly evaluated in *in vitro* fermentation of mud crab gut contents (Fig. 1A). In this treatment, the gut content was anaerobically incubated with 0.5% of carbohydrates. The results revealed that five kinds of carbohydrates (GOS, XOS, L-sorbose, xylitol, and RS) stimulated the formation of butyric acid compared with the control. Next, in the screening, all of these carbohydrates were used for assessing the butyrate-producing stimulation and the results showed that both GOS and RS gave greater amount of butyric acid than did XOS, L-sorbose, xylitol, and control in the cultures of mud crab contents (Fig. 1B), but there was not statistically significant (*P<0.05*). Therefore, the two carbohydrates GOS and RS induced a higher level of butyric acid were selected to conduct further studies.

**FIGURE 1** Pre-screening of different kinds of carbohydrates stimulating the production of butyric acid in the *in vitro* fermentation with gut contents of mud cab in the Culture I (A) and Culture II (B).

**REFERENCES**

Li, Z., Tran, N.T., Ji, P., Sun, Z., Wen, X., Li, S. (2019) Effects of prebiotic mixtures on growth performance, intestinal microbiota and immune response in juvenile chu's croaker, *Nibea coibor*. Fish Shellfish Immunol. 89, 564-573. doi: 10.1016/j.fsi.2019.04.025

Sato, T., Kusuhara, S., Yokoi, W., Ito, M., Miyazaki, K. (2017) Prebiotic potential of L-sorbose and xylitol in promoting the growth and metabolic activity of specific butyrate-producing bacteria in human fecal culture. FEMS Microbiol. Ecol. 93, fiw227. doi: 10.1093/femsec/fiw227
